# Hypothalamic microstructure and function are related to body mass, but not mental or cognitive abilities across the adult lifespan

**DOI:** 10.1007/s11357-022-00630-3

**Published:** 2022-07-27

**Authors:** Melanie Spindler, Christiane M. Thiel

**Affiliations:** 1 Biological Psychology, Department of Psychology, School of Medicine and Health Sciences, Carl Von Ossietzky Universität Oldenburg, 26129 Oldenburg, Germany; 2 Cluster of Excellence “Hearing4all”, Carl Von Ossietzky Universität Oldenburg, 26129 Oldenburg, Germany; 3 Research Centre Neurosensory Science, Carl Von Ossietzky Universität Oldenburg, 26129 Oldenburg, Germany

**Keywords:** Aging, Hypothalamus, Microstructure, Neurite orientation dispersion and density imaging, Functional connectivity, Body mass

## Abstract

Physical, mental, and cognitive resources are essential for healthy aging. Aging impacts on the structural integrity of various brain regions, including the hippocampus. Even though recent rodent studies hint towards a critical role of the hypothalamus, there is limited evidence on functional consequences of age-related changes of this region in humans. Given its central role in metabolic regulation and affective processing and its connections to the hippocampus, it is plausible that hypothalamic integrity and connectivity are associated with functional age-related decline. We used data of *n* = 369 participants (18–88 years) from the Cambridge Centre for Ageing and Neuroscience repository to determine functional impacts of potential changes in hypothalamic microstructure across the lifespan. First, we identified age-related changes in microstructure as a function of physical, mental, and cognitive health and compared those findings to changes in hippocampal microstructure. Second, we investigated the relationship of hypothalamic microstructure and resting-state functional connectivity and related those changes to age as well as physical health. Our results showed that hypothalamic microstructure is not affected by depressive symptoms (mental health), cognitive performance (cognitive health), and comparatively stable across the lifespan, but affected by body mass (physical health). Furthermore, body mass changes connectivity to limbic regions including the hippocampus, amygdala, and nucleus accumbens, suggesting functional alterations in the metabolic and reward systems. Our results demonstrate that hypothalamic structure and function are affected by body mass, focused on neural density and dispersion, but not inflammation. Still, observed effect sizes were small, encouraging detailed investigations of individual hypothalamic subunits.

**Supplementary Information:**

The online version contains supplementary material available at 10.1007/s11357-022-00630-3.

## Introduction

With the proportion of the elderly population increasing around the globe, it is one of today’s greatest challenges to promote healthy aging. Detrimental influences on successful aging include poor physical and cognitive ability, leading to reduced quality of life, mental health problems, and higher nursing intensity [1]. Poor cognitive and physical ability in aging and age-related diseases have been linked to brain microstructural changes including systemic inflammatory processes, reduced structural integrity, and iron accumulation [2–4]. In recent years, several studies in elderly human subjects with and without cognitive problems have shown a variety of microstructural changes in brain regions such as the hippocampus, amygdala, and medial temporal lobe [5–7].

For example, in the hippocampus, magnetization transfer ratio (MTR), a marker for myelin, was found to be reduced in Alzheimer’s disease [8]. Additionally, in a recent study by Solar et al. [9], decreased hippocampal fractional anisotropy (FA), and increased mean diffusivity (MD), measuring the directionality and magnitude of diffusion in tissue, were related to age, peaking around 30–35 years, and correlated with memory performance [9]. Furthermore, microstructural signs of aging in the hippocampus could be reduced by cognitive stimulation/environmental richness [10] and by physical exercise [11]. In addition, a physical exercise intervention increased hippocampal functional connectivity with the posterior cingulate cortex, which was related to better memory performance [12]. In general, dysregulation of the metabolic system through physical inactivity, obesity, or endocrine imbalances increases the risk for pathological aging conditions [13]. Overall, these findings highlight the link between physical and cognitive function in the hippocampus and age.

The hippocampus is involved in the metabolic system by inhibiting activity of the hypothalamic–pituitary–adrenal (HPA) axis. It provides a negative feedback regulation of glucocorticoid synthesis in the hypothalamus, which is the key player in central control of the metabolic system, including body homeostasis, water and food intake, and endocrine regulation. Additionally, through the HPA-axis, the hypothalamus is involved in psychosocial stress and mental health, for example the development of mood disorders [14]. The fornix, a major white matter bundle connecting the hypothalamus with the hippocampus and thalamus, is affected in cognitive impairment in age [15]. Therefore, it is conceivable that age-associated changes in hippocampal structure and function are related to alterations in hypothalamic signaling.

To date, there is no evidence on the role of the hypothalamus in humans in aging. Recent rodent studies, however, identified the hypothalamus also as a key player in the aging process [16–18], suggesting that it could be involved in alterations of endocrine and metabolic processes in age, leading to more wide-spread aging effects by inducing inflammatory signaling. Hence, if hypothalamic microstructure is compromised by physical health or metabolic dysfunction in age, it could possibly affect the brain aging process.

The present study thus aimed at examining the role of hypothalamic microstructure and function in age and different domains of healthy aging (physical, mental, cognitive). However, as the hypothalamus is located close to the third ventricle, microstructure quantified by the abovementioned diffusion metrics derived from conventional diffusion tensor images (DTI) are likely influenced by confounding cerebrospinal fluid (CSF). We utilize a multi-compartment model of diffusion (neurite orientation dispersion and density imaging (NODDI)) to take these influences into account and complement our results by the conventional DTI metrics to place them into the context of previous research.

We used data of a large representative sample aged 18 to 88 years, derived from the Cambridge Centre for Ageing and Neuroscience (CamCAN) repository [19, 20] and employed a multi-modal approach combining comprehensive structural (NODDI, [21, 22], DTI, MTR) and functional (resting-state functional connectivity (rs-FC)) magnetic resonance imaging (MRI).

First, we investigated age-associated changes in hypothalamic microstructure and compared it to the previously found changes in hippocampal microstructure in age. We tested the hypothesis that older age and obesity are related to degradation of both hypothalamic and hippocampal microstructure, in terms of decreased myelination (MTR) [8], neural integrity (orientation dispersion (OD), intracellular volume fraction (ICVF), FA [9], and increased inflammation (isotropic volume fraction (ISOVF), MD [9]. In addition, we assumed cognitive function to be related to hippocampal rather than hypothalamic microstructural integrity, and mental health to be associated with hypothalamic, and to a lesser extent with hippocampal microstructure. Second, we analyzed whether changes in microstructural integrity of the hypothalamus are associated with changes in its functional connectivity. In a last step, we aimed to answer the question, whether such changes in functional connectivity that are related to microstructural changes, are additionally influenced by age and physical health.

## Materials and methods

### Participants

**Fig. 1:**
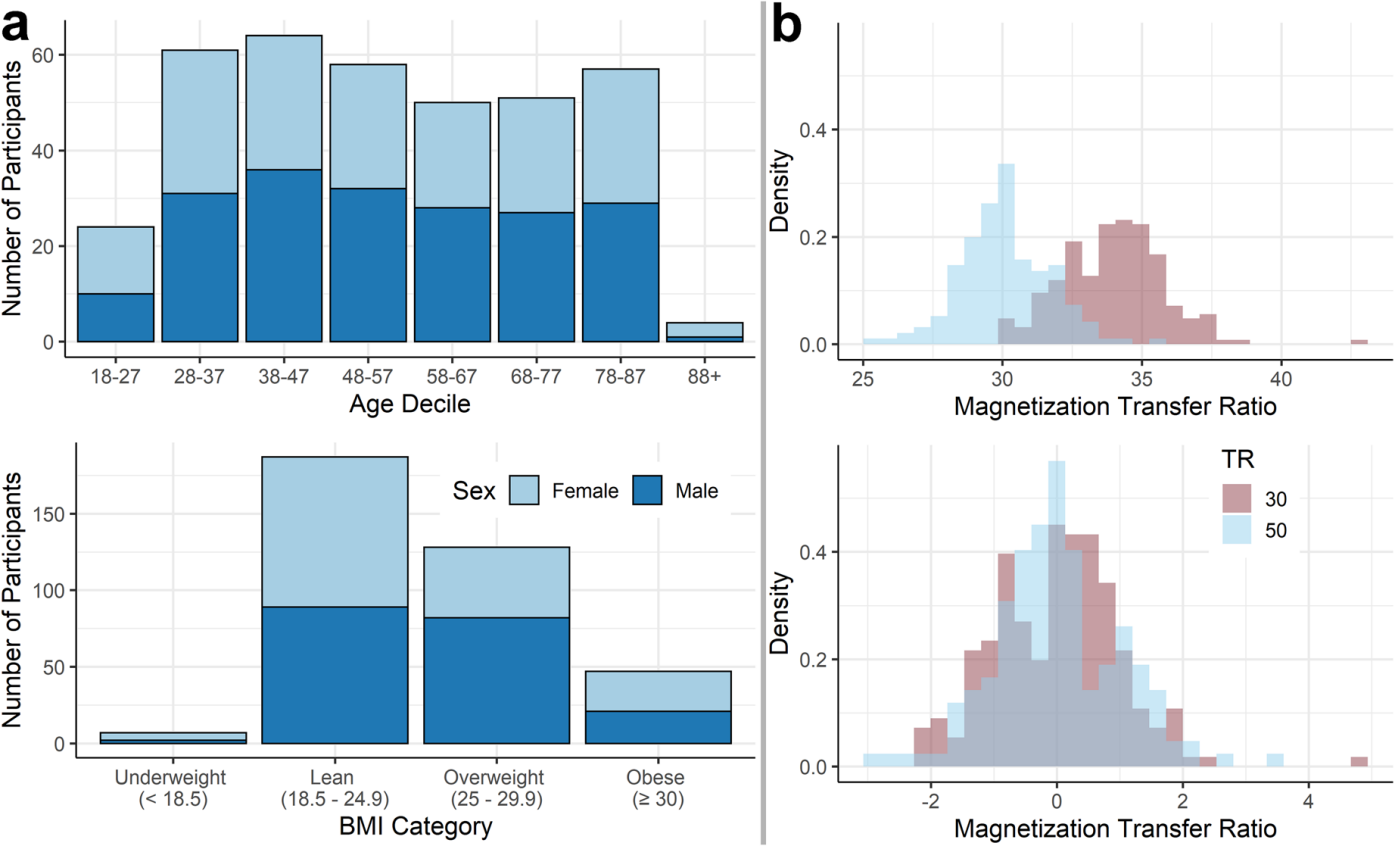
**a** Distribution of participants across sex and different age deciles (top) and body mass index categories (bottom, *n* = 369). **b** Density plot of magnetization transfer ratio values in the hypothalamus before (top) and after (bottom) standardization (*n* = 366)

Data were obtained from the CamCAN repository stage 2 datasets from the representative population-based sample (available at http://www.mrc-cbu.cam.ac.uk/datasets/camcan/) [19, 20]. Ethical approval was obtained from the Cambridgeshire 2 Research Ethics Committee, and participants gave written informed consent. For a sample description as well as inclusion and exclusion criteria for the CamCAN project, see Shafto et al. [19]. In short, participants had normal vision (at least 20/50 on the Snellen test) and hearing (able to hear 35 dB at 1000 Hz in either ear), basic cognitive abilities (Mini-Mental State Examination score >  = 24), and good knowledge of English language (native, or bilingual English speakers). Participants with self-reported substance abuse, major psychiatric conditions, current radiotherapy or chemotherapy, or a history of stroke were excluded. We employed additional exclusion criteria, including a history of psychiatric disease (e.g., depression needing treatment), diabetes, thyroid dysfunction, and left-handedness (assessed by the Edinburgh Handedness Inventory < 50) [23]. Furthermore, only participants who completed T1-weighted, magnetization transfer, resting state functional, and diffusion-weighted imaging were included in this study. After initial data review, one participant was excluded due to extremely large ventricles, resulting in a sample of *n* = 369 healthy participants (194 male, 175 female) aged between 18 and 88 years (Fig. 1a).

### Behavioral data selection

Further variables used from the dataset for the present analysis were education level, BMI (kg/m^2^), the Hamilton Anxiety and Depression scale scores (HADS-A/HADS-D) [24], and the total score of the Addenbrooke’s cognitive examination (ACE-R) [25]. The HADS-A and HADS-D are self-rating subscales comprised of seven questions each, rated between 0 and 3. The subscales aim at identifying symptoms of anxiety and depression during the preceding week.

The ACE-R is a test battery for mild cognitive impairment and dementia. It takes 10–15 min to complete [26], and is comprised of five domains: orientation/attention, memory, verbal fluency, language, and visuo-spatial. Taken together, a maximum total score of 100 points can be achieved. The BMI, HADS-A/HADS-D, and ACE-R scores were used as general indicators for physical, mental, and cognitive health, respectively, and were selected as measures that achieve comparable precision over the whole age range.

### MRI data acquisition

MRI data was acquired using a 3 T Siemens TIM Trio machine with a 32-channel head coil. During data acquisition, there was a change in gradient coil of the scanner. Whether data was acquired before or after, the change was added as a binary regressor in all analyses (coil change before vs. after). For each participant, a structural T1-weighted image was acquired with a magnetization-prepared gradient echo (MPRAGE) sequence, using a voxel size of 1 mm^3^, repetition time (TR) = 2250 ms, echo time (TE) = 2.99 ms, and a flip angle of 9°. Magnetization transfer images were acquired once at baseline and with radiofrequency pulse applied using a MT-prepared spoiled gradient sequence with 1.5 mm^3^ voxel resolution and a TE = 5 ms. The TR was either 30 ms or 50 ms in case the participants’ specific absorption rate stimulation limit was exceeded. For functional T2* images during rest (rs-fMRI), gradient echo planar imaging was conducted with the following parameters: TR = 1970 ms, TE = 30 ms, flip angle = 78°, voxel size of 3 × 3 × 4.44 mm (including a 20% gap: slice thickness 3.7 mm), and 261 volumes each with 32 slices in descending order. Participants were instructed to rest with their eyes closed. Diffusion MRI was obtained from a twice-refocused spin echo sequence, with 3 b_0_ volumes, and 30 directions of b_1000_ and b_2000_ volumes and the following parameters: 66 axial slices, 2 mm^3^ voxel resolution, TR = 9100 ms, and TE = 104 ms.

### MRI data processing

#### Segmentation

To obtain individual masks for the hypothalamus, the automated segmentation algorithm of Billot et al. [27] was used on the raw T1-weighted images, which resulted in five hypothalamic subunits per hemisphere. We binarized the images to obtain a mask for the whole hypothalamus. The T1-weighted images were segmented into tissue probability maps and registered to the SPM12 Montreal Neurological Institute (MNI ICBM152) template in 2 mm^3^ resolution [28] using trilinear interpolation. The same warps were then applied to the hypothalamus mask with nearest neighbor interpolation. A mask of the left and right hippocampus in MNI space was obtained using the Harvard–Oxford Subcortical Atlas with a threshold of 70 to ensure a reliable mask. Finally, all masks were visually inspected to ensure correct alignment and fit. In addition to raw T1-weighted images, raw diffusion-weighted images, raw rs-fMRI, and processed MTR maps were obtained from the CamCAN repository.

#### Microstructure

Hypothalamic and hippocampal microstructure was assessed using a three-compartment model of diffusion (NODDI) including intracellular, extracellular, and CSF components in each voxel. The derived parameters include ICVF, which is a measure for neurite density, i.e., the packing of neuronal tissue, OD, which represents the coherence of fibers, and ISOVF, which is a measure of free water (i.e., CSF). We complemented our analyses with MTR, a measure of myelination, and report conventional diffusion tensor parameters MD and FA, reflecting the magnitude (i.e., inflammation) and directionality (i.e., fiber integrity) of diffusion, respectively.

For that purpose, raw diffusion data were first denoised using MRtrix3 (dwidenoise) [29], and movement and eddy current corrected using FSL v.6.0.4 (eddy) [30]. The *b*-vectors were rotated accordingly and used together with the corrected data for all further analyses. On the one hand, the NODDI model was computed with the Accelerated Microstructure Imaging via Convex Optimization algorithm (AMICO, https://github.com/daducci/AMICO) [31], and on the other hand, the diffusion tensor was fitted on the *b*_1000_ shell data (dtifit). For MTR, the two images from the MT-prepared spoiled gradient sequence were coregistered and their ratio was computed. The obtained ICVF, OD, ISOVF, MTR, MD, and FA maps for each participant were then registered to the T1-weighted image, and subsequently non-linearly registered to 2 mm^3^ MNI space using the warp from native to MNI space (see “Segmentation”). Finally, we calculated mean hypothalamic and hippocampal ICVF, OD, ISOVF, MD, FA, and MTR for each participant using the masks obtained in “Segmentation” to quantify hypothalamic *microstructure.*

#### Functional connectivity

RS-fMRI data processing was performed in CONN [32, 33] based on the Statistical Parametric Mapping software package (SPM12, Wellcome Department of Imaging Neuroscience, London, UK) running on Matlab2019b (Mathworks Inc.). CONN’s default preprocessing pipeline included coregistration of the structural image to the mean functional image; segmentation of structural images into gray matter (GM), white matter (WM), and CSF; and subsequent normalization to MNI space. Total grey matter volume (GMV) was computed during segmentation. Functional data underwent realignment and unwarping, slice-time correction, and outlier detection based on the artifact detection tools (ART), normalization to MNI space [34], resampling to 2 mm^3^, and smoothing with a 5-mm Gaussian kernel. For normalization, structural and functional data were processed using the unified segmentation and normalization procedure in SPM12. The nonlinear transformations generated from the tissue probability maps in the segmentation were applied to the structural and functional data, respectively [34]. In a subsequent denoising step, the anatomical component-based noise correction procedure (aCompCor) was used to regress out movement parameters, BOLD signal from WM and CSF, outlier scans, and signal trends in the beginning of the session. Afterwards, a temporal band-pass filter between 0.008 and 0.09 Hz was applied to minimize the influence of remaining noise sources. To ensure data quality, subjects were excluded if the number of invalid/outlier scans detected with ART exceeded 30%. This resulted in seven subjects to be removed from the analyses (remaining *n* = 362 with 1.73% ± 3.67% outlier scans).

### Statistical analyses

For three participants, the TR used to obtain the magnetization transfer images was unknown. Therefore, these subjects were excluded from MTR analyses. Of the remaining participants, *n* = 208 were measured with TR = 30 ms, and *n* = 158 with TR = 50 ms. We assessed comparability of MTR values between the different TRs, and found that, throughout the brain, MTR was on average higher for images with TR = 30 compared to TR = 50. We therefore independently standardized hypothalamic and hippocampal MTR (0 ± 1) for all analyses separately for images obtained with a TR of 30 and 50, respectively (Fig. 1b).

#### Microstructure in healthy aging

To investigate the association of age, physical, mental, and cognitive health with hypothalamic and hippocampal microstructure, eight multiple regression analyses were performed using R v. 4.1.1 [35]. The models included ICVF, OD, ISOVF, and MTR of the hypothalamus and hippocampus as dependent variables and age, age^2^, BMI, sex, ACE-R score, HADS-D score, HADS-A score, GMV, and coil change during the study each as independent variables. To reduce multicollinearity induced by age and its quadratic term, the variable age was mean-corrected, and variance inflation factors (VIF) < 5 were considered acceptable. Bonferroni correction was employed to correct the significance level of each model for multiple comparisons (0.05/8 = 0.00625). For completeness, we report results of the same analysis using the tensor parameters MD and FA as outcome variables in the Online Resources Tab 1.

#### Microstructural influences on functional connectivity

To investigate the association between hypothalamic microstructure and functional connectivity, three multiple linear regression analyses with ICVF, OD, and ISOVF values were performed using the CONN toolbox [32, 33]. In the first level analysis (subject level), seed-to-voxel connectivity maps (Fisher-transformed correlation coefficients) using the average time-series of the hypothalamus ROI as a seed were generated. The resulting individual connectivity maps were entered into three-second level (i.e., group-level) regression analyses to assess changes in hypothalamic connectivity to the rest of the brain related to its microstructure. Here, the contrasts for rs-FC of the hypothalamus associated with hypothalamic ICVF, OD, and ISOVF, correcting for GMV and coil change, were investigated. To correct for multiple testing, the significance level was set to family-wise error (FWE) corrected cluster-level *p*_cluster_ < 0.017, and an uncorrected cluster forming threshold of *p*_voxel_ < 0.0003 on voxel-level (Bonferroni corrected for three analyses).

#### Functional connectivity in healthy aging

The final analyses were performed to investigate whether the changes in functional connectivity that are related to microstructural changes are additionally influenced by age, sex, and BMI. Two multiple linear regression analyses were performed using R v. 4.1.1 [35] to predict hypothalamic rs-FC with the hippocampus-amygdala region and the nucleus accumbens region. The models included age, sex, and BMI as predictors. The outcome variables were defined as the functional connectivity to the regions specified in “Microstructural Influences on Functional Connectivity”. To that aim, an overlap mask for the three contrasts in “Microstructural Influences on Functional Connectivity” was computed, comprised of the voxels significantly associated with hypothalamic ICVF, OD, and ISOVF. Then, the mean seed-to-voxel connectivity values of these regions and the hypothalamus were calculated and used for the two multiple linear regression analyses (hippocampus and amygdala; nucleus accumbens).

The sample size for these analyses was *n* = 362. VIF < 5 was considered acceptable. The models were considered significant with *p* < 0.025 after Bonferroni correction (0.05/2 = 0.025).

## Results

Demographic, behavioral, and physiological data of the study population is displayed in Table 1.

**Table 1:** Participant characteristics

Variable	Value	Range
Sex (male/female)	194/175	n.a
Education (0/1/2/3)^*^	22/49/61/237	n.a
Age in years (M ± SD)	54.1 ± 18.9	18–88
BMI in kg/m^2^ (M ± SD)	25.3 ± 4.0	17.4–45.4
HADS-D score (M ± SD)	2.4 ± 2.1	0–10
HADS-D category (0/1) †	357/11	n.a
HADS-A score (M ± SD)	4.5 ± 2.9	0–15
HADS-A category (0/1) †	319/49	n.a
ACE-R score (M ± SD)	95.3 ± 4.4	76–100
Coil change (before/after)	7/362	n.a

^*^Education was determined between 0 and 3, with 0 = none beyond the age of 16, 1 = GCSE/O-level, 2 = A-level, and 3 = university degree. ^†^Cutoff scores: 0 = 0–7; 1 = 8 or higher

### Microstructure in healthy aging

Eight multiple linear regression analyses were conducted to analyze the relationship between microstructure in the hippocampus and hypothalamus (each with ICVF, OD, ISOVF, and MTR as dependent variables) with age, age^2^, ACE-R score, BMI, sex, HADS-D and HADS-A scores, GMV, and coil change (independent variables). For age and GMV, the VIF was 4.6 and 4.7, respectively. For all remaining predictors, it was below 1.4.

**Fig. 2:**
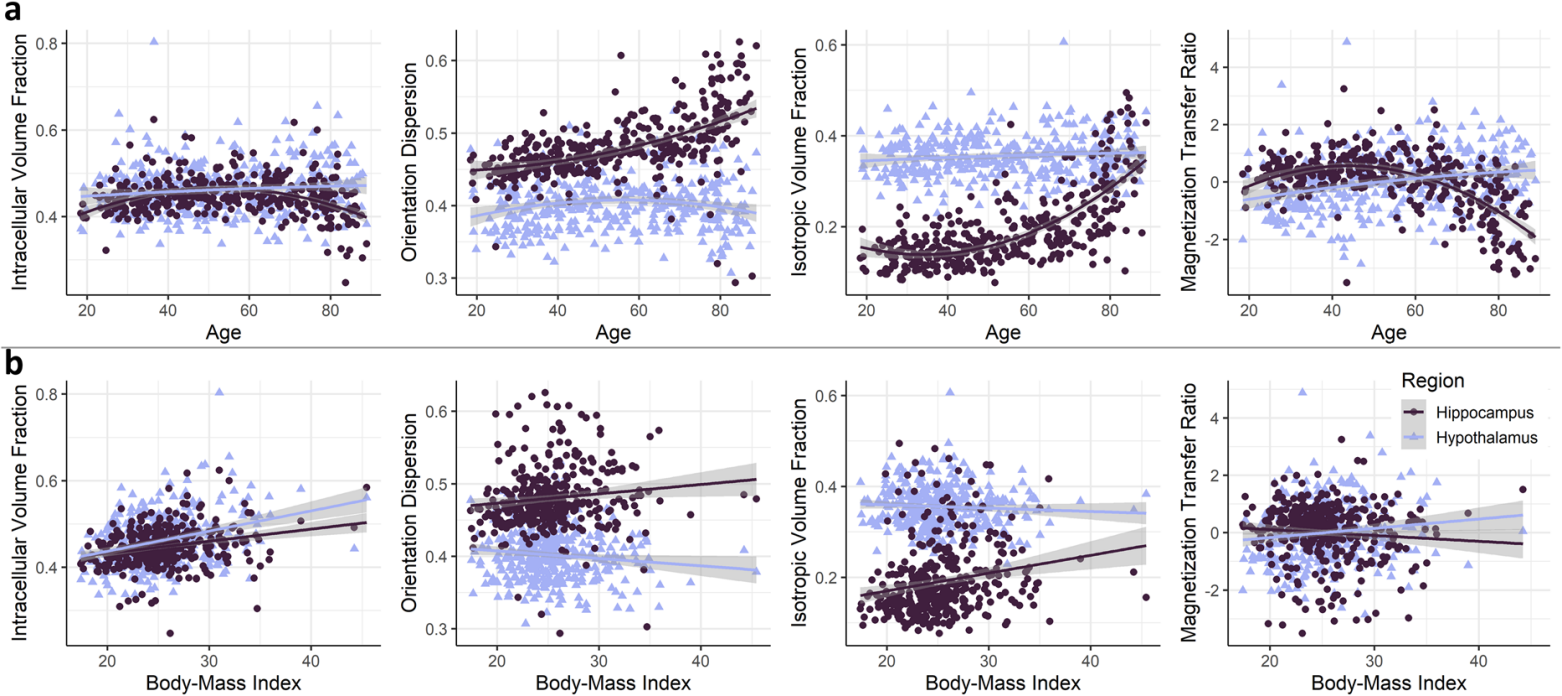
The association of **a** age and **b** body mass index (BMI) with microstructure of the hippocampus and hypothalamus, assessed by orientation dispersion (*n* = 369), isotropic volume fraction (*n* = 369), intracellular volume fraction (*n* = 369), and magnetization transfer ratio (*n* = 366). For the regression lines, a quadratic term was used

In the hippocampus (Table 2a), there was a significant association between all measures of microstructural integrity and age, suggesting a quadratic relationship (Fig. 2a). ICVF was significantly predicted by age, age^2^, sex, and BMI (adj. *R*^2^ = 0.254, *F*(9,357) = 14.84, *p* < 0.001), OD showed a significant association with age, age^2^, GMV, and sex (adj. *R*^2^ = 0.323, *F*(9,357) = 20.4, *p* < 0.001), ISOVF was associated with age, age^2^, and GMV (adj. *R*^2^ = 0.549, *F*(9,357) = 50.46, *p* < 0.001), and MTR was explained by age and age^2^ (adj. *R*^2^ = 0.405, *F*(9,354) = 28.41, *p* < 0.001), explaining between 25 and 55% of variance in hippocampal microstructure. With MTR and ICVF, a non-significant trend (*p* < 0.10) for the ACE-R score was observed.

**Table 2:** Results of the multiple regression analyses to predict microstructure (intracellular volume fraction (ICVF, *n* = 369), orientation dispersion (OD, *n* = 369), isotropic volume fraction (ISOVF, *n* = 369), and magnetization transfer ratio (MTR, *n* = 366)) of the hippocampus (a) and hypothalamus (b). Independent variables of interest included age, age^2^, psychological health (HADS-D, HADS-A), cognitive performance (ACE-R total score), and body mass index (BMI). To control for possible influences of sex, total gray matter volume (GMV), and coil change, these variables were introduced into the models as well

	a Hippocampus				b Hypothalamus			
**ICVF**	**Std.** ***β***	**SE**	***t***	***p***	**Std.** ***β***	**SE**	***t***	***p***
Intercept	− .645	.159	− 4.068	** < .001**	− .498	.172	− 2.890	**.004**
ACE-R	.090	.050	1.792	.074	.018	.055	0.333	.739
Age	− .010	.005	− 1.988	**.048**	.001	.006	0.152	.879
Age^2^	− .320	.047	− 6.834	** < .001**	.028	.051	0.542	.588
BMI	.282	.049	5.766	** < .001**	.312	.053	5.864	** < .001**
Coil	.182	.333	0.548	.584	.223	.362	0.615	.539
HADS-D	.002	.050	0.035	.972	.091	.055	1.657	.098
HADS-A	− .029	.053	− 0.542	.588	− .091	.058	− 1.571	.117
GMV	− .058	.098	− 0.596	.552	.047	.107	0.442	.658
Sex	.418	.100	4.197	** < .001**	.325	.108	3.005	**.003**
**OD**	**Std. *β***	**SE**	***t***	***p***	**Std. *β***	**SE**	***t***	***p***
Intercept	− .347	.151	− 2.299	**.022**	− .131	.180	− 0.731	.465
ACE-R	.014	.048	0.302	.763	.076	.057	1.337	.182
Age	.019	.005	3.811	** < .001**	< .001	.006	− 0.032	.974
Age^2^	.104	.045	2.326	**.021**	− .146	.053	− 2.744	**.006**
BMI	− .076	.047	− 1.642	.101	− .167	.055	− 3.005	**.003**
Coil	.337	.317	1.063	.288	− .230	.377	− 0.608	.543
HADS-D	.028	.048	0.592	.554	− .054	.057	− 0.951	.342
HADS-A	− .007	.051	0.140	.889	.020	.060	0.334	.739
GMV	− .234	.093	− 2.511	**.012**	− .164	.111	− 1.476	.141
Sex	.227	.095	2.395	**.017**	.092	.113	0.813	.417
**ISOVF**	**Std. *β***	**SE**	***t***	***p***	**Std. *β***	**SE**	***t***	***p***
Intercept	− .074	.123	− 0.597	.551	.300	.181	1.652	.100
ACE-R	− .025	.039	− 0.638	.524	.057	.057	0.985	.325
Age	.018	.004	4.587	** < .001**	− .003	.006	− 0.553	.580
Age^2^	.270	.036	7.407	** < .001**	− .024	.054	− 0.449	.654
BMI	− .030	.038	− 0.777	.437	− .126	.056	− 2.257	.025
Coil	.139	.259	0.538	.591	− .044	.381	− 0.116	.908
HADS-D	− .025	.039	− 0.629	.530	− .052	.057	− 0.912	.363
HADS-A	.006	.041	0.150	.881	< .001	.061	0.007	.994
GMV	− .361	.076	− 4.737	** < .001**	− .277	.112	− 2.470	.014
Sex	.049	.077	0.630	.529	− .196	.114	− 1.720	.086
**MTR**	**Std. *β***	**SE**	***t***	***p***	**Std. *β***	**SE**	***t***	***p***
Intercept	.196	.142	1.379	.169	.014	.177	0.077	.939
ACE-R	.076	.045	1.695	.091	− .036	.056	− 0.654	.513
Age	− .026	.005	− 5.621	** < .001**	.003	.006	0.533	.594
Age^2^	− .381	.042	− 9.112	** < .001**	− .046	.052	− 0.891	.373
BMI	.082	.046	1.789	.075	.003	.057	0.060	.952
Coil	− .110	.297	− 0.370	.712	.148	.370	0.402	.688
HADS-D	.020	.045	0.455	.649	.092	.056	1.637	.102
HADS-A	− .006	.048	− 0.130	.896	− .043	.059	− 0.726	.469
GMV	− .012	.088	− 0.136	.892	− .214	.110	− 1.948	.052
Sex	− .126	.089	− 1.417	.157	− .011	.111	− 0.095	.924

Significant results (*p* < .05) are marked in bold

In the hypothalamus (Table 2b), ICVF displayed a positive correlation with sex and BMI (adj. *R*^2^ = 0.118, *F*(9,357) = 6.422, *p* < 0.001), OD was negatively related to age^2^ and BMI (adj. *R*^2^ = 0.038, *F*(9,357) = 2.626, *p* < 0.005, Fig. 2b), and MTR showed a trend for a negative association with GMV (adj. *R*^2^ = 0.078, *F*(9,354) = 4.404, *p* < 0.001), explaining between 4 and 12% of variance in hypothalamic microstructure. With ICVF, a non-significant trend for the depression score was observed (*p* < 0.10). No significant association was found for ISOVF (adj. *R*^2^ = 0.022, *F*(9,357) = 1.897, *p* = 0.051). See Online Resource Tab.1. And Online Resource Fig. 1 for the predictions of hypothalamic and hippocampal FA and MD. To further investigate whether the influence of BMI on microstructural changes depends on age, we examined the interaction between age and BMI on hypothalamic ICVF, OD, and ISOVF (Online Resource Fig. 2). We found no hint towards a change of this relationship based on age.

### Microstructural influences on functional connectivity

**Fig. 3:**
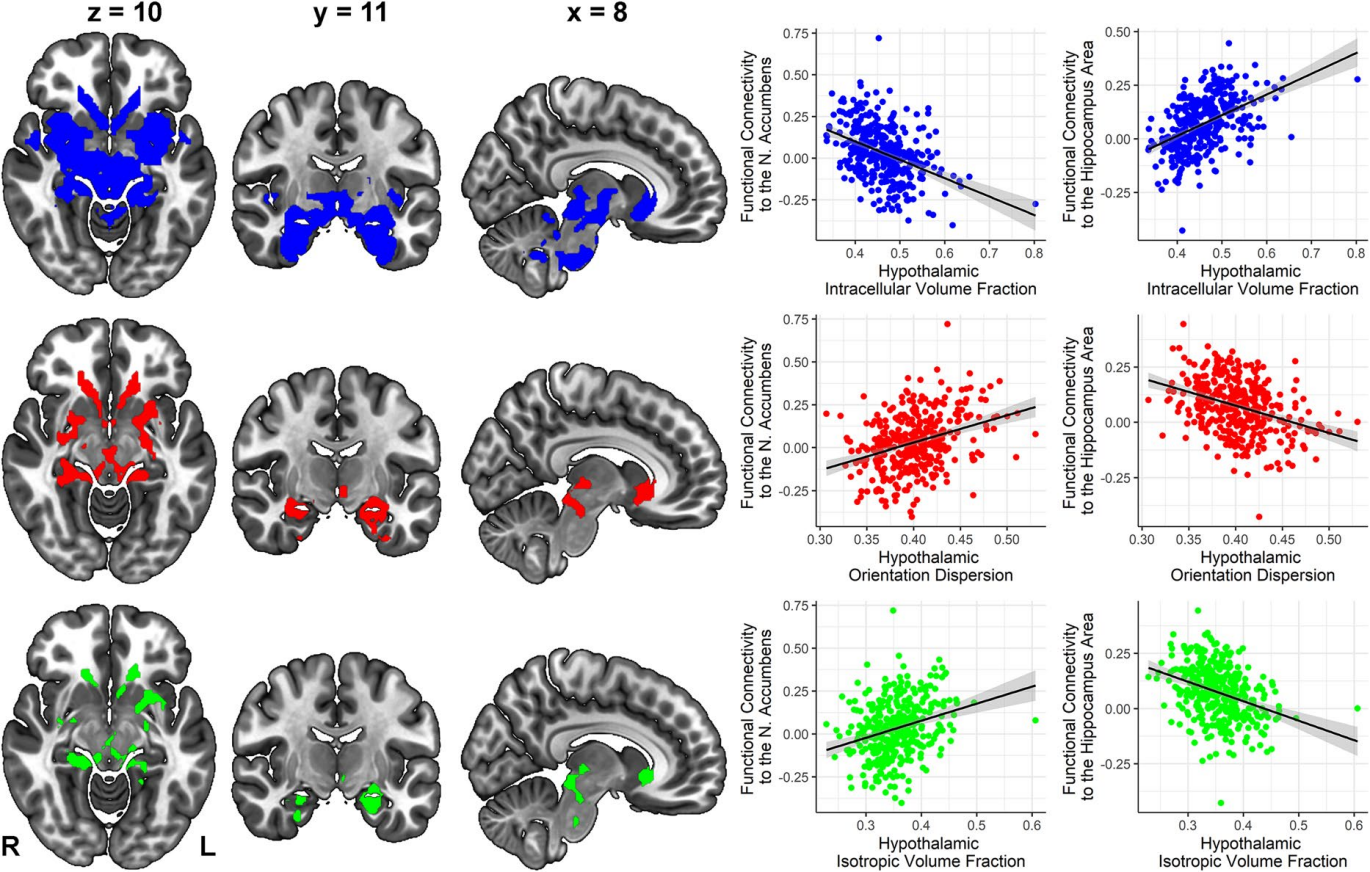
Resting-state functional connectivity of the hypothalamus as a function of hypothalamic microstructure (intracellular volume fraction, orientation dispersion, isotropic volume fraction), as determined by seed-based multiple regression analyses. The overlap of the clusters with all three parameters is displayed in triplanar view (*x* = 8, *y* = 11, *z* = 10, *n* = 362)

To assess how hypothalamic microstructural integrity is related to rs-FC of this brain region, three seed-based multiple linear regression functional connectivity analyses were performed. For each ICVF, OD, and ISOVF, we found a significant impact on hypothalamic functional connectivity to a cluster comprising the amygdala, hippocampus, and brainstem, as well as a cluster containing the nucleus accumbens and caudate (largest significant clusters for each contrast, *p*_cluster_ < 0.001). Here, OD and ISOVF were negatively correlated with functional connectivity of the hypothalamus to the hippocampus-amygdala regions, and ICVF was positively correlated with hypothalamic connectivity to these regions. In contrast, functional connectivity to the nucleus accumbens was positively associated with OD and ISOVF, and negatively with ICVF (Fig. 3). Hence, hypothalamic microstructural integrity was associated with functional connectivity changes of the hypothalamus with limbic brain regions (see Online Resource Table 2 for a complete list of significantly associated brain regions in these contrasts).

### Functional connectivity in healthy aging

Given that microstructural integrity of the hypothalamus is related to age, BMI, and sex on the one side as well as functional connectivity to limbic brain regions on the other side, the final analysis aimed to assess whether age, BMI, and sex also impact on rs-FC of the hypothalamus. To that aim, two multiple linear regressions were conducted with mean rs-FC to these regions as outcome variable. Age, BMI, and sex were used as predictor variables. The VIF was < 1.2 for all predictors.

**Fig. 4:**
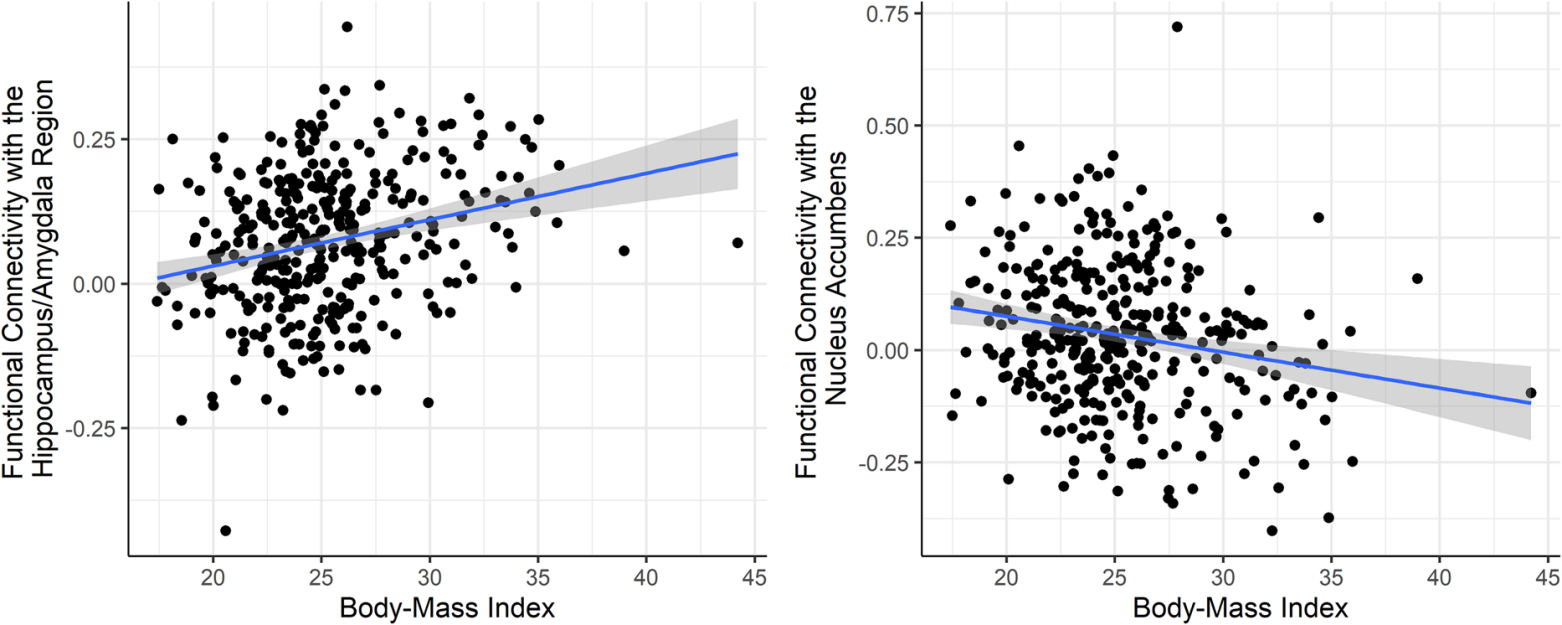
Scatterplot displaying the association of body mass index with hypothalamic resting-state functional connectivity to the hippocampus-amygdala region (left) and the nucleus accumbens (right)

In both models, BMI was associated with rs-FC to regions associated with hypothalamic microstructure, suggesting a positive relationship with the hippocampus-amygdala cluster, and a negative relationship with the nucleus accumbens cluster. No significant association was observed for sex or age, but a non-significant trend for a positive correlation of age with nucleus accumbens rs-FC (hippocampus-amygdala connectivity: adj. *R*^2^ = 0.065, *F*(4,358) = 9.333, *p* < *0.0*01; nucleus accumbens connectivity: adj. *R*^2^ = 0.039, *F*(4,358) = 5.818, *p* < 0.001). In other words, we found an additional, albeit small influence of BMI on functional connectivity of the hypothalamus, suggesting a stronger positive functional coupling with the hippocampus and amygdala, and stronger negative coupling with the nucleus accumbens with higher BMI (Fig. 4, Table 3).

**Table 3:** Results of two multiple linear regression analyses predicting functional connectivity of the hypothalamus with limbic regions with age, body mass index (BMI), and sex (*n* = 362)

a Hippocampus-amygdala connectivity	Std. *β*	SE	*t*	*p*
Intercept	.089	.164	− 0.545	.586
Age	− .086	.054	− 1.587	.113
BMI	.285	.054	5.233	** < .001**
Sex	.058	.102	.573	.567
b Nucleus accumbens connectivity				
Intercept	.130	.166	0.784	.434
Age	.097	.055	1.753	.080
BMI	− .224	.055	− 4.057	** < .001**
Sex	− .086	.103	− 0.825	.410

a, Functional connectivity with hippocampus and amygdala regions; b, Functional connectivity with the nucleus accumbens region

Significant results (*p* < .05) are marked in bold

## Discussion

To our knowledge, this is the first MRI study investigating the role of the hypothalamus in aging in humans. We combined comprehensive hypothalamic microstructure with functional connectivity in a large sample of healthy individuals between 18 and 88 years of the CamCAN repository (*n* = 369) and compared our findings to microstructural changes of the hippocampus, a region consistently associated with aging.

The data of our first analysis replicate and strengthen previous findings of altered microstructure in the hippocampus in older age [36, 37]. Consistent with prior literature, we observed a decrease in ICVF and MTR, measures of structural integrity and myelin, and an increase of OD [38, 39] and ISOVF in older age, which may be related to disorganization of white matter and inflammation, respectively [40, 41]. In early adulthood (approximately until the age of 40), age was associated with increased neural integrity, suggested by a quadratic relationship, confirming a recent study by Solar et al. [9]. For MTR and ICVF, we also found a tendency for an association between cognitive capabilities and hippocampal microstructure, which is in line with previous research [42].

Surprisingly, we observed hypothalamic microstructure to be stable across the lifespan. Only for OD, a reverse association with age was observed, such that older participants showed significantly lower OD compared to younger participants. This could be explained by decreased dendritic complexity throughout the aging process, which has been previously reported in different cortical areas in age as well [38]. Yet, the predictors were only able to explain a very small proportion of variance in hypothalamic microstructure, as compared to the hippocampus. The difference in age-associated changes in the hippocampus and hypothalamus could be explained by the mechanisms of neural plasticity in both structures. While the hippocampus is a highly myelinated region showing plasticity throughout the lifespan [43], the hypothalamus is an early-developing structure. Thus, it might be more resilient towards degeneration in age, an idea formulated in the last-in-first-out hypothesis of aging [44].

Nevertheless, research on rodents has found that in age, loss of neural stem cells and activation of microglial cells in the hypothalamus contribute to age effects, such as altered immune regulation (nuclear factor κB) and subsequent systemic inflammation (e.g., tumor necrosis factor α) [17, 45]. But to our knowledge, these inflammatory effects have mostly been measured indirectly (e.g., blood serum). On the voxel level, these aging effects could be limited to specific nuclei or subregions of the hypothalamus. This notion is supported by studies showing that across the lifespan, some cell groups in the hypothalamus decline, but others remain stable or even increase (e.g., corticotropin-releasing-hormone containing neurons in the paraventricular nucleus) [46]. Thus, studies employing high-resolution imaging techniques allowing for parcellation of the hypothalamus into subunits are necessary to investigate this relationship. Furthermore, potential aging effects on structural connectivity of the hypothalamus and associated brain regions remain to be explored.

In addition to age, we observed no significant effect of mental and cognitive health on hypothalamic microstructure, but demonstrated an effect of physical health, with increased BMI relating to higher hypothalamic ICVF and lower OD, complemented by higher FA and lower MD (Online Resources Fig.1). It is interesting to note that there was no significant relationship between BMI and ISOVF, suggesting that changes in MD observed in the hypothalamus might rather be driven by neural density and fiber coherence than water content/inflammation. This is contradictory to findings that suggest hypothalamic inflammation, indexed by MD, [47, 48] and gliosis [49] in obesity. Due to the comparatively low spatial resolution of standard diffusion-weighted sequences like that used in the CamCAN, the partial volume contaminations of CSF and white matter can only be controlled for to a limited extent and can highly influence the diffusion parameters derived from conventional tensor models (e.g., MD). Therefore, an advantage of this study is the utilization of a multi-compartment diffusion model to take the effects of different tissue types into account, and together with conventional DTI and MTR to allow better integration of the findings into the context of previous research.

Furthermore, we combined our structural findings with resting-state functional connectivity. Here, we aimed to disentangle the observed individual influences of age, physical health, and sex on hypothalamic microstructure and functional connectivity. We first identified brain regions whose functional connectivity to the hypothalamus was affected by changes in hypothalamic microstructure. We found that ICVF, OD, and ISOVF were all predictive of hypothalamic connectivity with two clusters comprising the amygdala, hippocampus, brainstem, and the nucleus accumbens and caudate. In the second step, we analyzed functional connectivity with these regions as a function of age, body mass, and sex to assess functional consequences of the link between alterations in hypothalamic microstructure and physiology. We found that BMI, but not age and sex, was predictive of hypothalamic functional connectivity with these clusters.

The amygdala, hippocampus, and nucleus accumbens are regions involved in the reward circuitry of the brain, and their connection to the (lateral) hypothalamus via dopaminergic pathways is well established [50, 51]. It would be possible that functional coupling between these areas and the hypothalamus is associated with alterations in the functional processing of food in obesity, possibly reflecting greater hedonic involvement. This is in line with previous research showing a positive correlation between BMI and activity of the hippocampus and amygdala during rest and presentation of high-caloric food cues [52]. In addition, signaling of the amygdala has been shown to predict the willingness to eat [53]. However, BMI was associated with stronger positive rs-FC between the hypothalamus and hippocampus-amygdala region, whereas it was related to stronger negative rs-FC between the hypothalamus and nucleus accumbens. This suggests that BMI differentially affects communication of the hypothalamus with connected reward-related brain regions, possibly associated with their individual roles within the reward circuit, for example, concerning the mechanisms of food wanting and liking [51].

Even though BMI is the most used indicator of physical health, it is an unspecific measure that can be influenced by both reduction in muscle mass and increase in white adipose tissue, occurring simultaneously in age. Thus, its role in aging is disputed [54]. Additional information such as energy expenditure, physical activity, waist circumference, or fat mass [1] could help shedding light onto the mechanisms of physical health-related microstructural changes in age. It is known that in older individuals, the metabolic system is more often confronted with diabetes, hypertension, muscle loss/loss of muscle function [55], thus increasing the risk of developing obesity. Therefore, in our study, effects of physical health and age are likely to overlap, hindering interpretation of the individual findings, which is a general problem that needs to be taken into consideration, as most indices of physical health decline with age [56]. Alternatively, it would be possible that the influence of physical health changes over the lifespan, but this idea could not be supported here (Online Resource Fig. 2).

Finally, it remains unclear how influences of age and physical health in the hypothalamus are temporally related, how one affects the other, and which other participant characteristics could further explain hypothalamic microstructure. For example, potential effects of sleep and hormonal medication (e.g., oral contraceptive use) need to be considered [57, 58]. Longitudinal investigations could shed light onto these mechanisms to better understand their individual and shared contributions to better target interventions aiming at healthy aging.

## Conclusion

To our knowledge, this study is the first to address the relations between hypothalamic microstructure and function in healthy aging in humans. In summary, we found hypothalamic microstructure and functional connectivity to remain relatively stable as a function of age, whereas they were predicted by body mass, suggesting changes in neural density and dendritic complexity. Furthermore, rs-FC of the hypothalamus with the amygdala, hippocampus, and nucleus accumbens was altered with increasing BMI. We argue that these connections could hint towards disturbed functioning of the dopaminergic reward circuit and its interaction with the metabolic system. For instance, reduced physical health affected rs-FC between the hypothalamus and amygdala, possibly reflecting stronger positive coupling of the metabolic and reward-related systems. Overall, our results show an impact of BMI on hypothalamus structure and function relationships, pointing towards alterations in neural density and dispersion, but not inflammation. Still, the effect sizes observed were small, highlighting the need for more detailed investigations of individual hypothalamic subregions.

## Supplementary Information

Below is the link to the electronic supplementary material.

Supplementary file1 (DOCX 628 KB)

## Data Availability

Raw and preprocessed data are available from the CamCAN repository (https://camcan-archive.mrc-cbu.cam.ac.uk/dataaccess/index.php). Analysis code and resting state analysis result files are available on the Open Science Framework: https://osf.io/qh9ga/
