# Supplementary material for: Hypothalamic microstructure and function are related to body mass, but not mental or cognitive abilities across the adult lifespan
Source: GeroScience. 2022 Jul 27;45(1):277–91. doi: 10.1007/s11357-022-00630-3 (PMC9886766; doi:10.1007/s11357-022-00630-3)
Supplement: Supplementary file 1 — Supplementary file1 (DOCX 628 KB) [file 11357_2022_630_MOESM1_ESM.docx]

**Supplementary Information: Hypothalamic microstructure and function are related to body mass, but not mental or cognitive abilities across the adult lifespan**

Melanie Spindler^1*^, Christiane M. Thiel^1,2,3^

University of Oldenburg

melanie.spindler@uol.de

In the hippocampus, fractional anisotropy was associated with age, age², BMI, and sex (Adj. R²=.273, F(9,357)=16.27, p<.001), and mean diffusivity was predicted by age, age², BMI, and GMV (Adj. R²=.421, F(9,357)=30.51, p<.001).

In the hypothalamus, fractional anisotropy was significantly associated with BMI (Adj. R²=0.059, F(9,357)=3.403, p<.001), with a non-significant trend for age² (p<.10). Mean diffusivity was significantly predicted by GMV and BMI (Adj. R²=0.057, F(9,357)=3.473, p<.001), with a trend for sex (p<.10).

**Tab. 1:** Results for the multiple linear regressions predicting hypothalamic and hippocampal fractional anisotropy (FA) and mean diffusivity (MD).

|  | a Hippocampus | | | | b Hypothalamus | | | |
| --- | --- | --- | --- | --- | --- | --- | --- | --- |
| FA | **Std. β** | **SE** | **t** | **p** | **Std. β** | **SE** | **t** | **p** |
| Intercept | -.545 | .156 | -3.494 | **<.001** | -.198 | .178 | -1.109 | .268 |
| ACE-R | .062 | .049 | 1.265 | .207 | -.022 | .056 | -0.381 | .703 |
| Age | -.023 | .005 | -4.617 | **<.001** | .001 | .006 | 0.113 | .910 |
| Age² | -.210 | .046 | -4.567 | **<.001** | .099 | .053 | 1.875 | .062 |
| BMI | .142 | .048 | 2.957 | **.003** | .266 | .055 | 4.829 | **<.001** |
| Coil | -.163 | .327 | -0.498 | .619 | -.160 | .374 | -0.428 | .669 |
| HADS-D | -.031 | .049 | -0.621 | .535 | .022 | .057 | 0.396 | .692 |
| HADS-A | .049 | .053 | 0.940 | .348 | -.056 | .060 | -0.928 | .354 |
| GMV | -.029 | .096 | -0.301 | .763 | .091 | .110 | 0.823 | .411 |
| Sex | .352 | .098 | 3.602 | **<.001** | .131 | .112 | 1.173 | .242 |
| MD | **Std. β** | **SE** | **t** | **p** | **Std. β** | **SE** | **t** | **p** |
| Intercept | .039 | .140 | 0.280 | .779 | .284 | .178 | 1.597 | .111 |
| ACE-R | -.053 | .044 | -1.192 | .234 | .050 | .056 | 0.888 | .375 |
| Age | .017 | .005 | 3.786 | **<.001** | .002 | .005 | 0.359 | .720 |
| Age² | .274 | .041 | 6.632 | **<.001** | .021 | .053 | 0.400 | .689 |
| BMI | -.146 | .043 | -3.379 | **<.001** | -.188 | .055 | -3.431 | **<.001** |
| Coil | .174 | .293 | 0.593 | .554 | -.063 | .374 | -0.170 | .865 |
| HADS-D | -.011 | .044 | -0.237 | .813 | -.086 | .056 | -1.532 | .126 |
| HADS-A | .016 | .047 | 0.348 | .728 | .072 | .060 | 1.209 | .227 |
| GMV | -.292 | .086 | -3.381 | **<.001** | -.268 | .110 | -2.436 | **.015** |
| Sex | -.026 | .088 | -0.291 | .771 | -.185 | .112 | -1.659 | .098 |


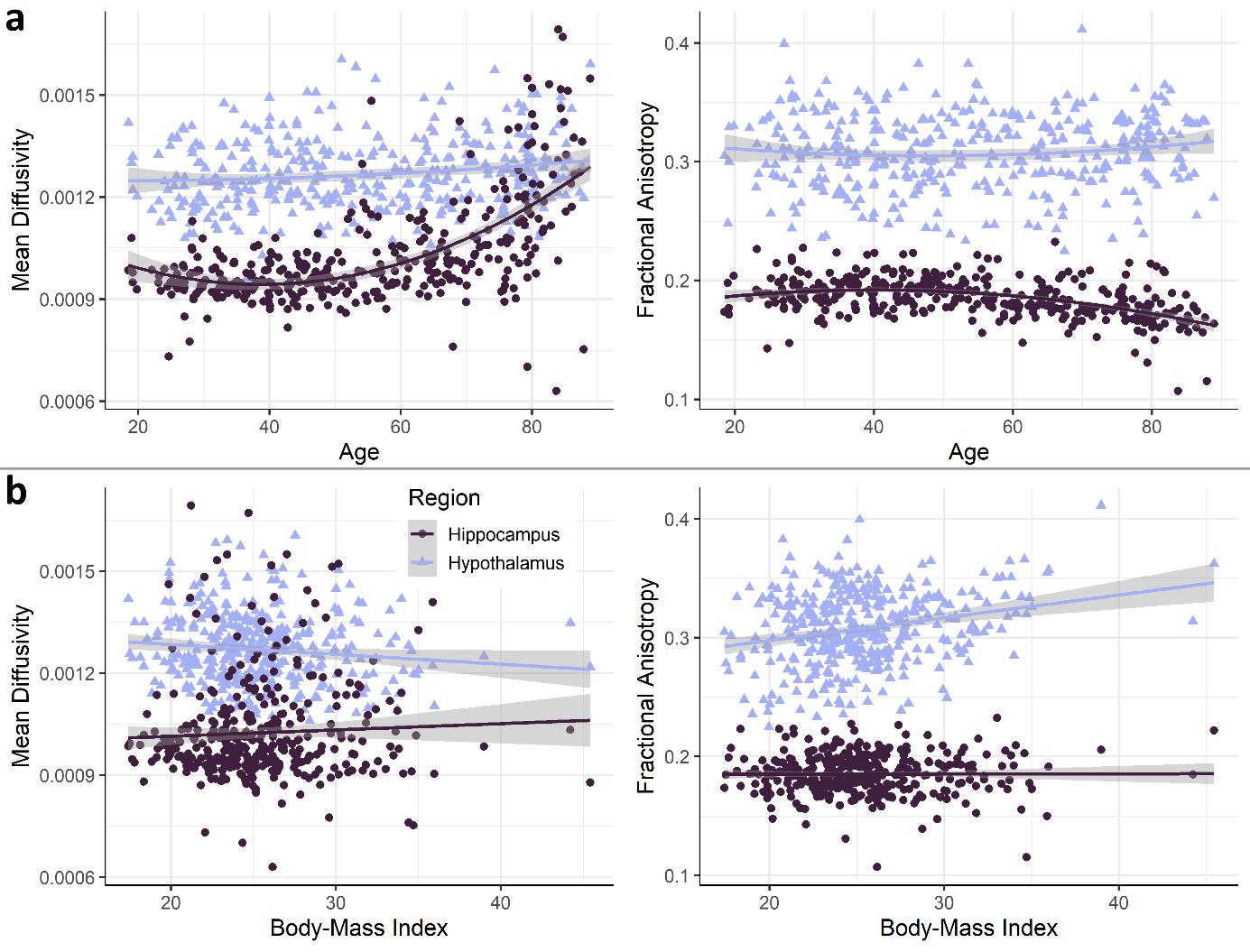


**Fig. 1:** Scatterplot showing the relationship between hypothalamic and hippocampal microstructure measured by fractional anisotropy and mean diffusivity and age and body-mass index.

**Tab. 2:** Peak MNI coordinates and corresponding brain regions for the multiple regression of functional connectivity with hypothalamic microstructure (hypothalamus as seed region, k>500).

| Region | Hemisphere | Size (voxel) | Peak MNI coordinate | | | Cluster size p_FWE_ |
| --- | --- | --- | --- | --- | --- | --- |
|  |  |  | x | y | z |  |
| A *Intracellular Volume Fraction* |  |  |  |  |  |  |
| Hippocampus, Brainstem, Amygdala | L+R | 13278 | +24 | -6 | -16 | <.001 |
| Cerebellum | L+R | 1835 | +14 | -76 | -52 | <.001 |
| Nucleus Accumbens, Caudate | L+R | 1520 | +10 | +22 | -6 | <.001 |
| Superior Parietal Lobe | L | 559 | -24 | -28 | +30 | <.001 |
| Frontal Pole | R | 534 | +22 | +44 | -24 | <.001 |
| *B Orientation Dispersion* |  |  |  |  |  |  |
| Hippocampus, Brainstem, Amygdala | L+R | 2740 | +24 | -6 | -16 | <.001 |
| Nucleus Accumbens, Caudate | L+R | 1089 | +16 | +28 | -8 | <.001 |
| *C Isotropic Volume Fraction* |  |  |  |  |  |  |
| Hippocampus, Brainstem, Amygdala | L+R | 2236 | -28 | +10 | -12 | <.001 |
| Nucleus Accumbens, Caudate | L+R | 618 | -8 | +18 | -4 | <.001 |


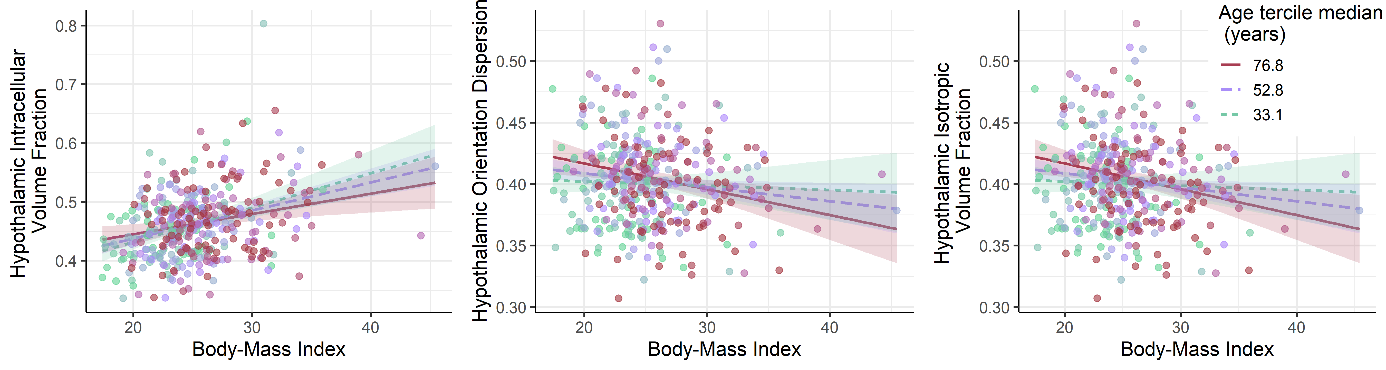


**Fig. 2:** Scatterplot examining a possible interaction effect of age and body-mass index (BMI) on hypothalamic microstructure (Left: Intracellular Volume Fraction, Middle: Orientation Dispersion, Right: Isotropic Volume Fraction), where the continuous variable age was divided into three terciles.
